# Riboflavin depletion promotes longevity and metabolic hormesis in *Caenorhabditis elegans*

**DOI:** 10.1111/acel.13718

**Published:** 2022-09-30

**Authors:** Armen Yerevanian, Luke M. Murphy, Sinclair Emans, Yifei Zhou, Fasih M. Ahsan, Daniel Baker, Sainan Li, Adebanjo Adedoja, Lucydalila Cedillo, Nicole L. Stuhr, Einstein Gnanatheepam, Khoi Dao, Mohit Jain, Sean P. Curran, Irene Georgakoudi, Alexander A. Soukas

**Affiliations:** ^1^ Department of Medicine, Diabetes Unit and Center for Genomic Medicine Massachusetts General Hospital Boston Massachusetts USA; ^2^ Department of Medicine Harvard Medical School Boston Massachusetts USA; ^3^ Leonard Davis School of Gerontology University of Southern California Los Angeles California USA; ^4^ Department of Biomedical Engineering Tufts University School of Engineering Medford Massachusetts USA; ^5^ Department of Medicine and Pharmacology University of California San Diego San Diego California USA; ^6^ Broad Institute of Harvard and MIT Cambridge Massachusetts USA

**Keywords:** AMPK, *C. elegans*, dietary restriction, FOXO, longevity, *rft‐1 riboflavin transporter*, riboflavin, UPR^mt^

## Abstract

Riboflavin is an essential cofactor in many enzymatic processes and in the production of flavin adenine dinucleotide (FAD). Here, we report that the partial depletion of riboflavin through knockdown of the *C. elegans* riboflavin transporter 1 (*rft‐1*) promotes metabolic health by reducing intracellular flavin concentrations. Knockdown of *rft‐1* significantly increases lifespan in a manner dependent upon AMP‐activated protein kinase (AMPK)/*aak‐2*, the mitochondrial unfolded protein response, and FOXO/*daf‐16*. Riboflavin depletion promotes altered energetic and redox states and increases adiposity, independent of lifespan genetic dependencies. Riboflavin‐depleted animals also exhibit the activation of caloric restriction reporters without any reduction in caloric intake. Our findings indicate that riboflavin depletion activates an integrated hormetic response that promotes lifespan and healthspan in *C. elegans*.

## Graphical abstract

Riboflavin depletion via knockdown of the *C. elegans* riboflavin transporter *rft‐1* promotes longevity and metabolic hormesis. Riboflavin depletion mimics features of dietary restriction, and lifespan extension occurs through the activation of AMPK, FOXO, and the mitochondrial unfolded protein response.

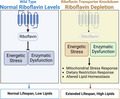

**Abbreviations**
AMPK: AMP‐activated protein kinase
ATP: adenosine tri‐phosphate
ETC: electron transport chain
EV: empty vector
FAD: flavin adenine dinucleotide
FLIM: fluorescence lifetime imaging
FMN: flavin mononucleotide
FOXA: forkhead box A
FOXO: forkhead box Group O
GC/MS: gas chromatography/mass spectrometry
LB: Luria Broth
LC/MS: liquid chromatography/mass spectrometry
NAD(P)H: nicotinamide adenine dinucleotide phosphate
NGM: nematode growth medium
QPCR: quantitative PCR
RNAi: RNA interference
SRS: stimulated Raman scattering
TOR: target of rapamycin
TORC1: TOR complex 1
TORC2: TOR complex 2
TPEF: two‐photon excited fluorescence
UPRmt: mitochondrial unfolded protein response

## 1 INTRODUCTION

Healthy mitochondrial function requires the coordination of multiple cellular inputs including sufficient energetic substrates, amino acids, and micronutrients. Vitamin cofactors are key to metabolic processes such as the citric acid cycle, electron transport chain, and energy shuttling into the cytosol. One family of vitamins that participate in mitochondrial physiology are the flavins (Mansoorabadi et al., 2007). The flavin co‐factors include flavin mononucleotide (FMN) and flavin adenine dinucleotide (FAD) and are essential for redox chemistry and electron shuttling (Massey, 1995). FAD is classically known to serve as an electron acceptor in the conversion of succinate to fumarate by succinate dehydrogenase in the citric acid cycle, as well as an electron donor to complex II of the electron transport chain. The flavins are also cofactors for multiple enzyme classes including the oxidoreductases and the fatty acid dehydrogenases (Lienhart et al., 2013). They are derived from riboflavin, a water soluble ribitol derivative also known as vitamin B2. Riboflavin is an essential nutrient for all animals and must be acquired either from food sources or from commensal gut flora (Powers, 2003).

The animal kingdom has evolved specific transporters to import riboflavin from the gut lumen and to transport them intracellularly (Moriyama, 2011). In humans and mice, three isoforms of these transporters are expressed by three distinct genes: Slc52A1, Slc52A2, and Slc52A3 (Subramanian et al., 2011; Yamamoto et al., 2009; Yonezawa et al., 2008). Disruption in the function of these transporters is known to induce pathology through cellular riboflavin depletion (Nabokina et al., 2012). Congenital deficiency in these transporters is associated with clinical syndromes in humans including Brown‐Vialetto‐Van Laere syndrome, where patients experience progressive neurologic deficits, and is treated successfully with extremely large doses of riboflavin (Dipti et al., 2005; Spagnoli & De Sousa, 2012).

The *C. elegans* genome harbors two riboflavin transporter orthologs, *rft‐1* and *rft‐2* (Biswas et al., 2013). Previous work has shown that the riboflavin transporters have differential expression and phenotypes based on knockdown. (Biswas et al., 2013). *rft‐1* expression is localized to the intestine and knockdown leads to a complete elimination of brood size. *rft‐2* expression occurs in the pharynx and intestine and is associated with reduced brood size. While the loss of function of *C. elegans* orthologs of riboflavin transporters is known to lead to embryonic lethality, it is not known whether the depletion of riboflavin after early embryonic and larval development has deleterious consequences. Based upon published literature, riboflavin deficiency is predicted to have broad metabolic impacts on the organism, including reducing cellular respiration by impacting the citric acid cycle and mitochondrial electron transport activity, as well as reducing the enzymatic function of a wide variety of oxidoreductase enzymes important for anabolic activity. It remains unknown whether the impact of such broad metabolic perturbations following development are deleterious, or whether there are advantageous aspects based upon the activation of energetic stress responses under conditions of low riboflavin and FAD levels. Hormetic responses and extended lifespan due to energetic disruption are a well described phenomenon in *C. elegans* (Dillin et al., 2002; Feng et al., 2001). The traditional focus on cofactor biology has been that increasing micronutrient intake provides beneficial effects to the organism. We took the contrarian view that like macronutrient restriction, micronutrient restriction, under the right circumstances, could produce beneficial effects by creating energetic stress or through other means, with riboflavin being an obvious candidate given its prominent role in metabolism.

In the present study, we ask whether flavin co‐factor depletion via disrupting the normal uptake of riboflavin can promote advantageous, metabolic stress defenses such as those activated by dietary restriction and mitochondrial energetic stress. We determine that physiologic riboflavin depletion alters cellular energetics and activates key longevity factors AMPK, FOXO, and the mitochondrial unfolded protein response (UPR^mt^). Riboflavin depletion via riboflavin transporter knockdown extends lifespan and promotes healthspan in *C. elegans*, dependent upon an AMPK‐UPR^mt^‐FOXO signaling relay. These data suggest that riboflavin depletion provides metabolic benefits and by leveraging factors mobilized by caloric restriction and energetic stress without actual reductions in caloric intake. Further, despite the deleterious impacts of monogenic diseases in riboflavin transporters, selective knockdown of transporters, and alteration of riboflavin physiology may provide translational opportunities to manage energetic states and thus metabolic diseases of aging.

## 2 RESULTS

### 2.1 Knockdown of *rft‐1* promotes longevity via riboflavin depletion

**FIGURE 1:**
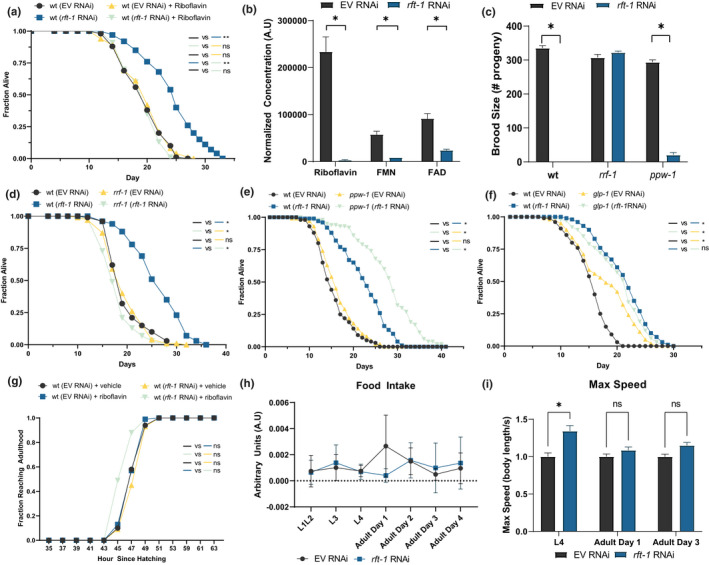
*rft‐1* RNAi depletes flavins and extends lifespan. (a) Knockdown of the riboflavin transporter *rft‐1* via RNA interference significantly extends lifespan in wild type (wt) animals versus empty vector RNAi (EV). Addition of 665 μM riboflavin abrogates this lifespan extension (see Table S1 for tabular data and replicates). (b) LC/MS analysis of worm lysates collected at adult day 1 treated with *rft‐1* RNAi reveals significant reductions in organismal riboflavin, FMN, and FAD concentrations. (c) Brood size is diminished in *rft‐1* RNAi treated animals and remains suppressed in *ppw‐1* animals (somatic RNAi competent, germline RNAi incompetent) but is rescued in *rrf‐1* animals (somatic RNAi blunted, germline RNAi competent), suggesting a somatic site of *rft‐1* action. (d) *rrf‐1* animals exhibit lifespans equivalent to wt on *rft‐1* RNAi. (e) *ppw‐1* mutants experience lifespan extension with *rft‐1* RNAi. (f) Germline deficient *glp‐1* animals (kept at the non‐permissive temperature, 25°C) do not experience additional lifespan extension with *rft‐1* RNAi. (g) Developmental rate from the first larval stage to adulthood is unchanged in animals treated with *rft‐1* RNAi, with or without riboflavin. (h) Food intake for animals treated with EV and *rft‐1* RNAi is not different across development and adulthood. (i) Maximum speed levels are higher in L4 and intact for adult day 1 and day 3 animals treated with *rft‐1* RNAi compared to controls. For lifespans results represent at least two biological replicates. See Table S1 for tabular data and replicates of survival analyses. *Indicates *p* < 0.05, **, *p* < 0.001 by log‐rank analysis (a through g), standard two‐way ANOVA (b,c,h,i). Bars represent means ± SEM. NS, non‐significant

The lack of brood and prominent intestinal expression suggested that *rft‐1* had the most prominent metabolic phenotype, so, we examined consequences of its knockdown (Gandhimathi et al., 2015). *rft‐1* depletion via RNA interference (RNAi) prompts a significant, 25% increase in lifespan (Figure 1a). RNAi knockdown of *rft‐1* reduces the transporter's mRNA by approximately 70%, suggesting that partial riboflavin transporter deficiency rather than complete knockdown induces this phenotype. (Figure S1a). mRNA encoding the paralogous riboflavin transporter *rft‐2* trends non‐significantly upward with *rft‐1* RNAi, which indicates that there is not significant compensation for *rft‐1* knockdown by *rft‐2*, and confirms the specificity of the RNAi‐based knockdown of *rft‐1* (Figure S1a) To confirm that the longevity phenotype of the *rft‐1* knockdown animals is due to reduced riboflavin uptake rather than a non‐canonical effect of the transporter, we administered high dose riboflavin supplementation to attempt to overcome the deficit in transport. As expected, high doses of riboflavin abrogate the lifespan increase attributable to *rft‐1* RNAi (Figure 1a). This strongly suggests that riboflavin is the etiologic factor in the *rft‐1* RNAi phenotype, and further that the depletion of riboflavin due to transporter deficiency is the source of lifespan extension. Worm lysates of young adult worms treated with *rft‐1* RNAi exhibit marked reductions in riboflavin, FMN and FAD levels as assessed by quantitative liquid chromatography/mass spectrometry (LC/MS) (Figure 1b). In parallel, we evaluated the expression of enzymes key to flavin co‐enzyme synthesis including riboflavin kinase (*rfk‐1*) and FAD synthetase (*flad‐1*). *rft‐1* RNAi was not associated with reductions in expression of *flad‐1* and *rfk‐1* which produce FAD and FMN, respectively, suggesting stoichiometric depletion of FMN/FAD rather than reductions in the enzymes that govern production (Figure S1a).

Previous descriptions of brood size deficits in *rft‐1* knockdown animals suggests that the germline may play a role in the phenotype (Biswas et al., 2013; Qi et al., 2017; Yen et al., 2020). We utilized *rrf‐1* (somatic RNAi blunted, germline RNAi competent) and *ppw‐*1 (somatic RNAi competent, germline RNAi incompetent) mutants and examined brood size and lifespan on *rft‐1* RNAi. The loss of brood size is dependent on somatic action of *rft‐1* (i.e., normalized in *rrf‐1* mutants which do not efficiently conduct somatic RNAi). This suggests that a somatic process is altering metabolic and reproductive capacity (Figure 1c). Lifespan was also dependent on somatic action of *rft‐1*, as *rrf‐1* mutants did not exhibit lifespan extension on *rft‐1* RNAi (Figure 1d). Germline loss of RNAi extended lifespan further on *rft‐1*, suggesting that preserved germline uptake of riboflavin accentuates somatic depletion of riboflavin and potentiates the lifespan extension (Figure 1e). Lifespan extension with *rft‐1* RNAi in a long‐lived *glp‐1* mutant lacking germline stem cells is significantly blunted, suggesting that the germline is required to act as a riboflavin sink for beneficial effects of *rft‐1* RNAi to manifest (Figure 1f). The reduction in brood size raised the question of whether germ line stem cells and oocyte production are altered by riboflavin depletion. We examined the presence of the germline stem cells and oocytes via DAPI staining, which revealed an intact germline and oocyte production (Figure S1b). Animals treated with *rft‐1* RNAi exhibit normal developmental timing (Figure 1g), indicating that delays in development do not play a role in lifespan extension with *rft‐1* knockdown.

In order to quantify the temporal dynamics of somatic riboflavin‐related gene expression as a proxy for flavin biology across the lifespan, we examined the expression of riboflavin transporter and FAD synthetic enzymes in germlineless *glp‐4* mutants. Somatic *rft‐1* and *rft‐2* mRNAs increase in *glp‐4* animals at day 4 but return back to normal levels through aging on day 10. *Rfk* and *flad‐1* expression increase through the life of animals (Figure S1c). In spite of this, *rft‐1* RNAi‐prompted lifespan extension is dependent upon whole life RNAi, as post‐developmental RNAi fails to extend lifespan (Figure S1d). The most probable explanation for this observation is that *rft‐1* knockdown very likely needs to be manifest during L4 to young adult development when rapid germline expansion is operative to effectively “steal” and deplete somatic riboflavin levels.

Riboflavin deficiency is associated with neurologic sequelae in mammals, and we wanted to verify that feeding behavior and motility is unchanged compared to control animals (Qi et al., 2017). The knockdown of *rft‐1* does not affect food intake as measured up to adult day 3 by a food disappearance assay (Figure 1h). Pharyngeal pumping rates are also unchanged. (Figure S1e). Max crawling speed increases with *rft‐1* RNAi (vs. vector control) in L4 larvae but is similar between adult day 1 and adult day 3 animals (Figure 1i). Average crawling speed shows similar patterns (Figure S1f). Animals spend equivalent amounts of time on food at L4 and adult day 1 when treated with vector vs. *rft‐1* RNAi (Figure S1g). Animal length and width were also similar across the lifespan. (Figure S1h) These findings suggest that animals treated with *rft‐1* knockdown exhibit normal developmental rate to adulthood, robust eating and size, and healthy activity levels in adulthood suggesting that the nutritional depletion of riboflavin does not reduce healthspan to achieve lifespan extension.

### 2.2 Riboflavin depletion promotes longevity through FOXO

**FIGURE 2:**
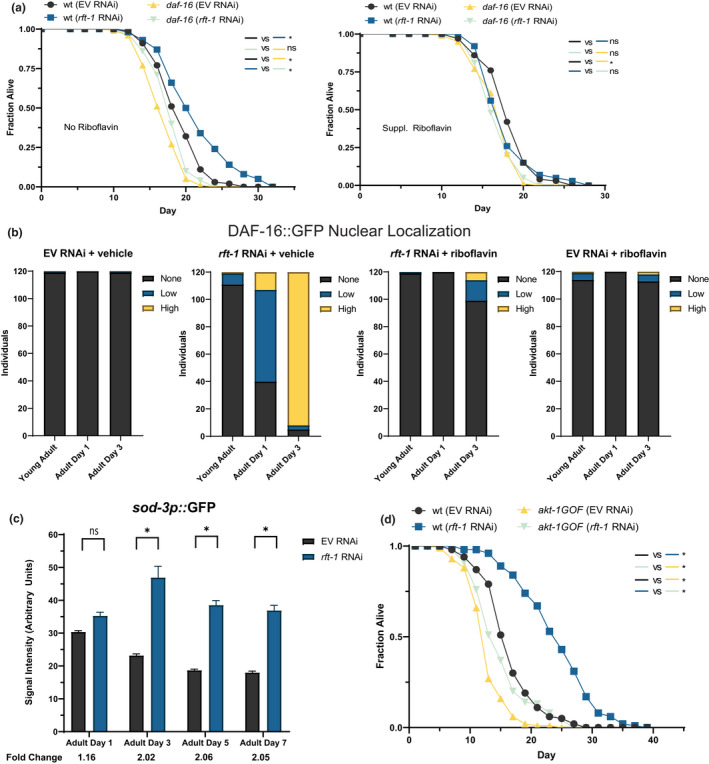
Riboflavin depletion promotes longevity by activating FOXO/*daf‐16*. (a) Lifespan extension with knockdown of the *rft‐1* transporter is abrogated in *daf‐16* mutants, with and without supplemental riboflavin. (b) Nuclear localization of DAF‐16 was increased following *rft‐1* RNAi in a riboflavin‐dependent manner as assessed by a DAF‐16::GFP translational reporter. Animals were binned into no nuclear localization, low levels of localization, and high levels of localization (see Figure S2 for representative images). N = 120 animals per condition, representative of two biological replicates. (c) A *sod‐3p*::GFP transcriptional reporter indicates increased activity of DAF‐16 significantly over the lifespan of *rft‐1* RNAi treated animals. (d) A chromosomally located *akt‐1* gain‐of‐function mutation blunts the longevity response to riboflavin depletion vs. wt. For a‐d, results are representative of at least two biological replicates. *Indicates *p* < 0.05 by log‐rank analysis (a and d), and by two‐way ANOVA followed by Sidak's multiple comparisons post‐hoc test (c). See Table S1 for tabular data and replicates of survival analyses. Bars represent means ± SEM

FOXO is known to act genetically downstream of nutrient‐sending manipulations that extend lifespan (Greer et al., 2007; Lee et al., 2003) and we hypothesized that it may be activated in the context of riboflavin depletion. Indeed, the loss of function in the sole *C. elegans* FOXO ortholog, *daf‐16*, is epistatic to lifespan extension with *rft‐1* RNAi, with or without the presence of supplemental riboflavin (Figure 2a). A DAF‐16::GFP translational reporter demonstrates greater nuclear localization at adult day 1 and day 3 with riboflavin depletion, suggesting that *rft‐1* RNAi activates DAF‐16 (Figure 2b and S2a). Transgenic DAF‐16::GFP worms treated with empty vector and *rft‐1* RNAi as synchronous L1 were subsequently transferred at adult day 1 to plates with and without riboflavin. By adult day 3, additional riboflavin completely abrogates the DAF‐16 nuclear localization evident in in *rft‐1* RNAi treated animals (Figure S2b). This indicates that the consequences of riboflavin depletion on *daf‐16* nuclear localization are reversible in adulthood. Confirming increased transcriptional activity of DAF‐16 in the setting of riboflavin depletion, RNAi of *rft‐1* leads to the upregulation of a *sod‐3p*::GFP reporter (Figure 2c). This activation is present through adult day 7, indicating consistent FOXO activation post‐developmentally (Figure S2c).

The activation of FOXO suggests either suppression of insulin‐like/PI‐3 kinase signaling (canonical FOXO activation) or activation via another mechanism. We examined whether insulin signaling was playing a role by examining the effect of *rft‐1* RNAi on *akt‐1* and *pdk‐1* gain of function mutants (Paradis & Ruvkun, 1998). These animals are short lived due to constitutive inhibition of FOXO. The *akt‐1* gain‐of‐function mutation abrogates lifespan extension attributable to *rft‐1* RNAi (Figure 2d). The *pdk‐1* gain‐of‐function mutant on *rft‐1* RNAi, conversely, still exhibits lifespan extension (Figure S2d). This indicates that the suppression of DAF‐16 activity via augmented Akt signaling abrogates lifespan extension prompted by riboflavin deficiency.

### 2.3 Riboflavin depletion alters cellular redox ratio and energetics

**FIGURE 3:**
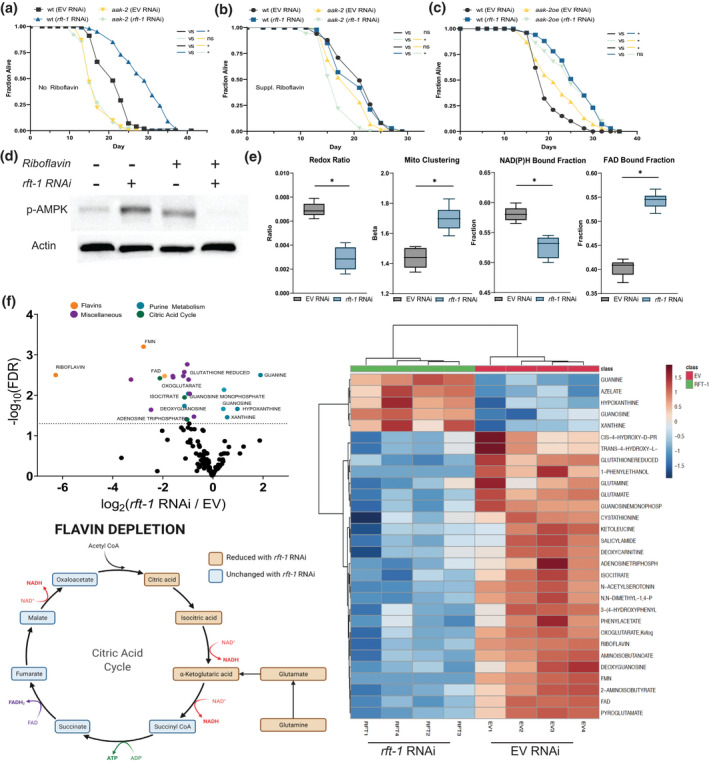
Riboflavin depletion alters cellular energetics. (a) Lifespan extension with *rft‐1* RNAi is abrogated in AMPK/*aak‐2* mutants. (b) Addition of riboflavin has a deleterious effect on lifespan in *aak‐2* mutants. (c) Lifespan extension with *rft‐1* RNAi is preserved in animals with the overexpression of *aak‐2* subunit aa 1–321 (*aak‐2oe*) (d) Western blotting of phospho‐AMPK^Thr172^ in lysates collected from young adult animals indicates activation following RNAi to *rft‐1*, an effect abrogated by the addition of riboflavin. (e) Box plots of results from two‐photon and fluorescence lifetime imaging, including organismal redox ratio, mitochondrial clustering, NAD(P)H bound fraction, and FAD bound fraction for EV and *rft‐1* RNAi treated animals. Riboflavin depletion decreases the redox ratio and increases intestinal mitochondrial clustering and the FAD bound fraction. (f) Volcano plot and heatmap of differentially abundant metabolites quantified by LC/MS reveals reductions in citric acid metabolites including citric acid, isocitric acid, and α‐ketoglutarate following riboflavin depletion. Purine metabolites including xanthine, hypoxanthine, and guanosine are enriched with *rft‐1* RNAi. Representation of citric acid metabolites impacted by riboflavin depletion. See Table S1 for tabular data and replicates of survival analyses. For (a–d) results are representative of two biological replicates. For (e), results are from 8 worms of two biological replicates. *Indicates *p* < 0.01 by log‐rank analysis (a–c) and by two tailed *t*‐test (e). Box and whisker plots (e) indicate median and 5/95th percentile, respectively. See Table S1 for tabular data and replicates of survival analyses

We suspected that AMPK may be similarly activated by energetic stress with riboflavin deficiency, and further that AMPK activation may be mechanistically linked to lifespan extension with *rft‐1* RNAi. Knockdown of *rft‐1* fails to promote longevity with loss of function in the AMPKα catalytic subunit *aak‐2* (Figure 3a), and this pattern is not altered by the addition of riboflavin (Figure 3b). Lifespan is still extended in long‐lived *aak‐2oe* animals with *rft‐1* RNAi (versus the same animals on vector RNAi control), suggesting that *aak‐2* is necessary but not sufficient for lifespan extension (Figure 3c). Consistent with AMPK activation by riboflavin depletion, phospho‐AMPK^T172^ levels are increased in *rft‐1* RNAi treated animals, and this effect is abrogated by the addition of riboflavin (Figure 3d). In order to determine whether AMPK is required for the activation of DAF‐16 under riboflavin depletion, we examined DAF‐16::GFP nuclear localization with and without functional *aak‐2*. Under *rft‐1* RNAi conditions, DAF‐16 nuclear localization still increases in the absence of *aak‐2* (Figure S3a).

The activation of AMPK suggests that modulation of cellular energetics might play a role in the longevity phenotype seen with the *rft‐1* knockdown. We hypothesized that reductions in flavin cofactor (FAD, FMN) concentrations induce mitochondrial stress responses due to the changes in organellar energetics by altering redox state. We examined the impact of riboflavin depletion on the redox ratio utilizing label‐free multiphoton microscopy and fluorescence lifetime imaging (FLIM) of intestinal cells in control and *rft‐1* RNAi treated animals (Figure S3b,c). Animals treated with *rft‐1* RNAi versus empty vector control‐treated animals exhibit a significant decrease in the optical redox ratio, defined as the ratio of FAD/(NAD[P]H + FAD) calculated based on the autofluorescence signatures of the corresponding co‐enzymes. There is also an increase in mitochondrial clustering, suggesting altered mitochondrial energetics in an oxidized state and morphologic changes to the mitochondrial network (Figure 3e) (Liu et al., 2018). A significant decrease in the NAD(P)H protein bound fraction suggests decreased levels of glutaminolysis and enhanced utilization of the glutathione pathway (Alonzo et al., 2016). An increase in the FAD bound fraction suggested that overall FAD depletion was causing aggressive capture of flavin cofactors by enzymatic machinery (Figure 3e). LC/MS metabolomics of *rft‐1* RNAi treated animals indicates significant changes in multiple metabolic pathways. Increases in purine catabolism metabolites are present, including xanthine, hypoxanthine, and guanosine (Figure 3f). Pathway enrichment analysis reveals that other than riboflavin metabolism, riboflavin deficiency leads to significant impact on metabolites in the glutathione and purine metabolic pathways (Figure S3d). Components of the citric acid cycle including citrate, isocitrate, and α‐ketoglutarate, as well as ATP, are reduced by riboflavin depletion. Glutamate and glutamine levels are also reduced suggestive of disruptions in glutamine synthesis (Figure 3f).

### 2.4 Riboflavin depletion activates the mitochondrial unfolded protein response

**FIGURE 4:**
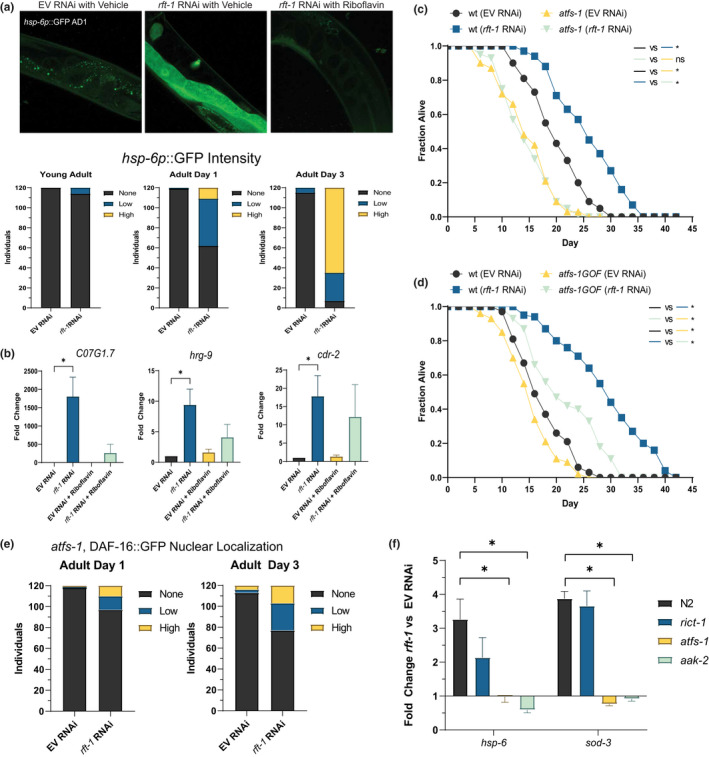
Activation of the mitochondrial unfolded protein response (UPR^mt^) is required for riboflavin depletion to promote longevity. (a) RNAi to *rft‐1* promotes activation of *hsp‐6*p::GFP progressively on adult days 1 and 3, an effect reversed by riboflavin supplementation (for binning, *N* = 120 worms per condition, representative of two biological replicates) Images above at 40×, binning performed at 10× magnification. See Figure S4 for binning examples. (b) Quantitative RT‐PCR of established *atfs‐1* target genes indicates marked upregulation with riboflavin depletion. (c) *atfs‐1* loss of function mutants do not experience lifespan extension with riboflavin depletion. (d) Gain of function mutants in *atfs‐1* are short lived but preserve responsiveness to *rft‐1* RNAi. (e) *tfs‐1*;DAF::16GFP animals exhibit decreased but present nuclear localization on *rft‐1* RNAi at both adult days 1 and 3. *N* = 120 animals. (f) QPCR of *hsp‐6* and *sod‐3* exhibit upregulation of transcripts in wild type animals treated with *rft‐1* RNAi and loss of upregulation in *atfs‐1* and *aak‐2* mutants. For (b,f), results are representative of at least three biological replicates. See Table S1 for tabular data and replicates of survival analyses. * and ** indicate *p* < 0.01 by two‐way ANOVA of ΔCt values (b,f), and by log‐rank analysis (c,d). (e) representative of population of 60 worms from two biological replicates. Bars represent means ± SEM

The frank changes in energetic status, altered redox ratio, and presence of mitochondrial clustering all suggested that mitochondrial stress responses may also be contributing to the longevity response to riboflavin deficiency. Indeed, the UPR^mt^ is activated by *rft‐1* knockdown, as evidenced by induction of *hsp‐6p*::GFP (Kimura et al., 2007) on days 1 and 3 of adulthood, and this effect is abrogated by the addition of riboflavin (Figure 4a). Full activation of the UPR^mt^ is known to require the transcription factor ATFS‐1, which translocates to the nucleus to activate stress response pathways (Wu et al., 2018). Established target genes of ATFS‐1, including *cdr‐2*, *hrg‐9*, and *C07G1.7* (Soo & Van Raamsdonk, 2021) are upregulated with *rft‐1* knockdown, with the previously undescribed ATFS‐1 target gene C07G1.7 exhibiting a 2000‐fold increase (Figure 4b). The UPR^mt^ activation is necessary for lifespan extension, as a*tfs‐1* loss of function animals, which have lower lifespans than wild‐type at baseline, do not exhibit lifespan extension with *rft‐1* knockdown (Figure 4c). In the setting of gain‐of‐function mutations in *atfs‐1*, which lead to shortened lifespan (Bennett et al., 2014), *rft‐1* knockdown still promotes significant extension of lifespan (Figure 4d). This indicates that the UPR^mt^ response is necessary but not sufficient for the riboflavin depletion longevity phenotype. We wished to evaluate the relationship between the UPR^mt^ and DAF‐16 and examined nuclear localization of DAF‐16 in *atfs‐1* mutants. *rft‐1* RNAi in *atfs‐1*;DAF‐16::GFP animals revealed abrogated nuclear localization, suggesting that DAF‐16 activation is partially dependent on *atfs‐1* and the UPR^mt^ (Figure 4e). We evaluated *sod‐3* and *hsp‐6* expression in *aak‐2* and *atfs‐1* animals to determine if the transcriptional responses to *daf‐16* and *atfs‐1* were present in these mutant strains with riboflavin depletion. QPCR revealed that the upregulation of *hsp‐6* and *sod‐3* with *rft‐1* RNAi are both dependent upon both *atfs‐1* and *aak‐2* (Figure 4f).

### 2.5 Riboflavin depletion alters somatic lipid stores

**FIGURE 5:**
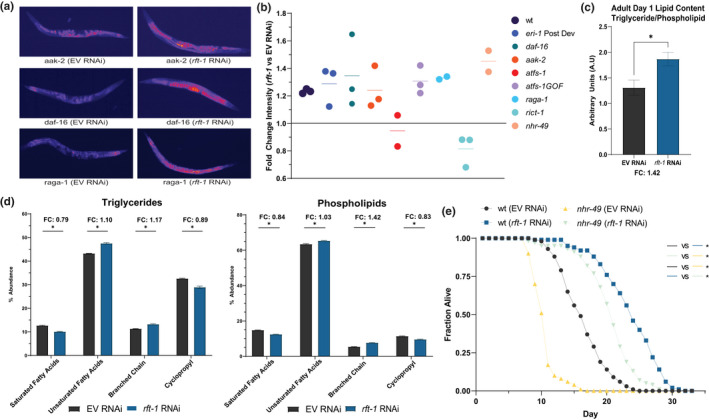
Riboflavin depletion alters lipid metabolism. (a) Fixative‐based nile red staining reveals marked upregulation of lipids in both soma and germline of *aak‐2*, *daf‐16* and *raga‐1* animals (b) Quantification of images and fold change differences between EV and *rft‐1* RNAi reveal significant increases in fat mass with multiple mutants including a post developmental *rft‐1* RNAi in *eri‐1* enhanced RNAi mutants and with larval exposure to *rft‐1* RNAi in multiple mutants including *daf‐16, aak‐2, nhr‐49, atfs‐1(GOF)*, and *raga‐1*. *atfs‐1* and *rict‐1* loss‐of‐function mutants do not exhibit increased fat on *rft‐1* RNAi. (c) Lipid analysis via GC/MS reveals an increase in overall fat stores (triglyceride/phospholipid ratio). (d) Shifts towards unsaturated fatty acid side chains and branched chain lipids in phospholipid and triglyceride fractions in aggregate (e) *nhr‐49* mutants exhibit intact lifespan extension with *rft‐1* RNAi. * indicates *p* < 0.05 by students 2‐tailed *t*‐test (c) and by two‐way ANOVA (d) and by log rank analysis (e). Bars (c,d) represent means ± SEM. Dots in (b) represent individual biological replicates as fold change values over EV treated animals

The long‐lived phenotype of riboflavin depletion and the role of flavin cofactors in beta‐oxidation suggests that changes in lipid composition may be manifest following *rft‐1* knockdown. We hypothesized that the changes in lipid metabolism occur upstream of or in parallel to FOXO activation, due to changes in enzymatic function (such as reduced activity of lipid dehydrogenases). RNAi of *rft‐1* induces significant increases in fat mass in both the intestine and the germline in adult day 1 worms, as exhibited by fixative‐based oil‐red‐O and nile red staining (Figure 5a, S5a).

We examined the epistatic relationships of the UPRmt, FOXO, and AMPK with regard to this high lipid phenotype. We also evaluated whether key lipid regulating pathways such as target of rapamycin (TOR) and *nhr‐49* play an important role in the lipid biology of riboflavin depletion. Epistasis analysis indicates that *rft‐1* RNAi increases fat mass in *daf‐16*, *aak‐2, nhr‐49,* and *raga‐1* animals but not in *atfs‐1* or *rict‐1* animals by fixative‐based nile red staining (Figure 5b). Post‐developmental *rft‐1* RNAi beginning at young adult stage in enhanced RNAi *eri‐1* animals also increases fat mass, indicating that riboflavin deficiency does not impact lipid metabolism exclusively through a developmental pleiotropy (Figure 5b). Confirming these observations and further delineating the nature of the lipids increased in abundance following *rft‐1* RNAi, stimulated Raman scattering (SRS) analysis of live adult day 1 animals indicates increased total signal of unsaturated fatty acids and the unsaturated to total lipid ratio in riboflavin deficiency (Figure S5b) (Nieva et al., 2012; Potcoava et al., 2014). By gas chromatography/mass spectrometry (GC/MS) of triglyceride and phospholipid fractions separated by solid phase extraction, global triglyceride stores increase by 40% in both young adult and adult day 1 *rft‐1* RNAi‐treated animals, consistent with the spectroscopic imaging and fixative‐based lipid staining (Figure 5c). While only small changes are evident by young adulthood (Figure S5c), by adult day 1 animals exhibit significant differences in their lipid composition, with increases in unsaturated and branched chain fatty acids, and reductions in cyclopropyl fatty acids in both triglyceride and phospholipid fractions (Figure 5d, S5c). Due to its role in lipid oxidation, we examined whether *rft‐1* lifespan extension was dependent upon *nhr‐49*. *rft‐1* RNAi significantly extends the lifespan of *nhr‐49* mutants (Figure 5e). Due to increases in branched chain fatty acid synthesis, we examined the expression of *acdh‐1*, which is a known branched chain dehydrogenase in *C.* *elegans* and that has been previously reported as a dietary sensor (Watson et al., 2013). An *acdh‐1* promoter GFP reporter is significantly increased ~70% with *rft‐1* RNAi at adult day 1 (Figure S5d). In order to begin to determine whether unsaturated fatty acids are elevated in riboflavin deficiency owing to increased production vs utilization, we examined the expression of the fatty acid desaturase *fat‐7* (Han et al., 2017). Riboflavin depletion does not promote changes in *fat‐7* expression early in life but preserves it with aging (Figure S5e).

### 2.6 Riboflavin depletion activates dietary restriction pathways

**FIGURE 6:**
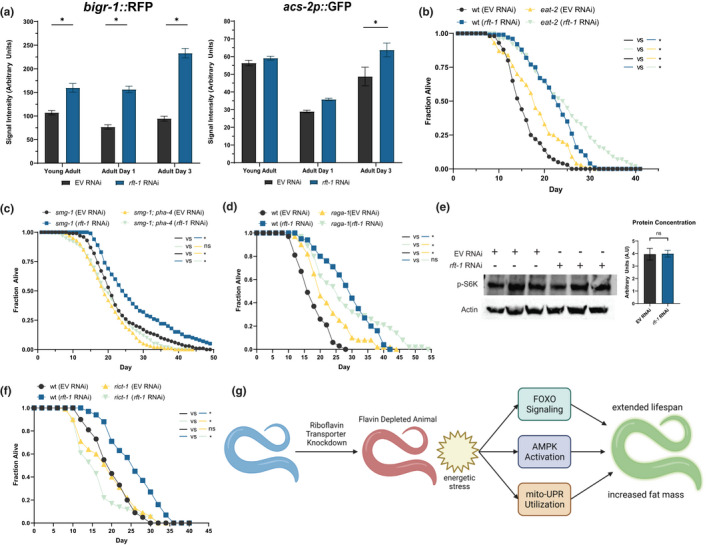
Riboflavin depletion mimics features of dietary restriction. (a) Imaging of reporters known to be activated with dietary restriction (*bigr‐1*::RFP, *right*, and *acs‐2p*::GFP, *left*) indicates activation under *rft‐1* RNAi that progresses with age. (b) Lifespan extension with *rft‐1* RNAi occurs in *eat‐2* mutants. (c) Lifespan extension is abrogated in *pha‐4* loss‐of function mutants versus temperature sensitive *smg‐1* mutant controls. (d) TORC1 mutant *raga‐1* exhibits lifespan extension with *rft‐1* RNAi. (e) Western blot of adult day 1 animals containing humanized S6K (permitting Western blotting for TORC1‐mediated phosphorylation of S6K^T389^) reveals no change in phospho‐S6K concentrations between control and riboflavin depleted worms. (f) TORC2 mutant *rict‐1* does not exhibit lifespan extension with riboflavin depletion. (g) Model of riboflavin depletion impact on metabolism and longevity. Knockdown of the riboflavin transporter leads to depletion of flavin co‐factors, influencing the energetic status of the animal and affecting global enzymatic activities dependent on FAD and FMN. This creates an integrated metabolic signaling response that promotes increased triglyceride stores and extends lifespan. See Table S1 for tabular data and replicates of survival analyses. *Indicates *p* < 0.05 by two‐way ANOVA (a), students *T*‐test (e), and log‐rank test (b–d). Bars represent means ± SEM

The long‐lived phenotype of riboflavin depletion, concomitant with decreases in energetics, AMPK activation, and impairment in lipid beta‐oxidation, suggested to us that riboflavin depletion mimics some features of a dietary restriction‐like phenotype. The *acs‐2* and *bigr‐1* genes are well established to be transcriptionally upregulated during periods of caloric restriction in *C. elegans* (Van Gilst et al., 2005; Wu et al., 2016). *rft‐1* RNAi induced *bigr‐1*::RFP and *acs‐2p*::GFP expression with age (Figure 6a). We sought to assess whether other canonical caloric restriction factors and processes were involved in riboflavin depletion. We examined *eat‐2* animals, which have extended lifespan owing to defective pharyngeal pumping, and noted that the animals experience further lifespan extension with *rft‐1* RNAi (Figure 6b) We also examined the *C. elegans* forkhead box A (FOXA) homolog *pha‐4,* known to be epistatic to caloric restriction‐mediated longevity (Panowski et al., 2007). Lifespan extension with *rft‐1* RNAi is dependent on *pha‐4*/*FOXA* (Figure 6c). Inhibition of TOR signaling is also important in the response to dietary restriction. Thus, we examined whether mutants in the TOR complex 1 (TORC1) and TOR complex 2 (TORC2) pathways exhibit longevity with riboflavin depletion. RAGA/*raga‐1* mutants, which have defects in TORC1 activation, experience lifespan extension on *rft‐1* RNAi (Figure 6d). To further determine whether altered TORC1 activity is required for the hormetic effects of riboflavin depletion, we used a strain of *C.* *elegans* that contains a knock‐in, humanized S6K, which permits immunoblotting for phospho‐S6K to determine the activity of TORC1. No difference is evident in phospho‐S6K between *rft‐1* RNAi and empty vector control, suggesting that altered TORC1 signaling is not essential to the biological response to riboflavin depletion (Figure 6e). In contrast, the loss of function mutations in the essential TORC2 subunit *rict‐1* experience no lifespan extension with *rft‐1* RNAi, indicating that riboflavin depletion requires TORC2 activity to exact its favorable effects (Figure 6f).

## 3 DISCUSSION

Vitamins, as essential cofactors for life, have traditionally been viewed as highly beneficial entities independent of their concentrations. This is particularly true of the B‐vitamins, which are water soluble and do not exhibit significant toxicities at moderate supraphysiologic doses. Our work in *C. elegans* counters the notion that more is always better, as depletion of key enzymatic co‐factor riboflavin can trigger metabolic and physiologic stress responses that are hormetic in nature and extend lifespan.

We were initially concerned that the reduction of the flavin cofactors such as FAD and FMN would be frankly toxic to the organism. This was particularly true when LC/MS revealed that FAD levels in the *rft‐1* treated animals were 80%–90% below normal. We anticipated that the loss of FAD to this level would prevent the function of succinate dehydrogenase and the ability of the electron transport chain (ETC) to absorb electrons produced by the citric acid cycle. Our data does indeed suggest disruptions in mitochondrial respiration, with decreased redox ratios, mitochondrial clustering, reduced ATP production, and activation of the UPR^mt^. Unexpectedly, however, riboflavin depletion has lifespan dependencies that differ from of classic ETC disruption, such as in *cco‐1* and *frh‐1* (frataxin) knockdowns (Durieux et al., 2011; Schiavi et al., 2013). Previous examinations of *cco‐1* and *frh‐1* RNAi have shown that lifespan extension with ETC disruption is AMPK and FOXO independent (Durieux et al., 2011, Schiavi et al., 2013) and *atfs‐1* independent (Bennett et al., 2014). These epistatic relationships to lifespan extension suggest a different “flavor” of UPR^mt^, and that riboflavin depletion does not represent a solitary poisoning of the ETC as seen through complex I–IV knockout or knockdown. Riboflavin depletion is likely having pleiotropic effects leading to alternative ways of activating UPR^mt^ (i.e., enzymatic disruption), and that the function of the UPR^mt^ under these circumstances may be managing stress responses that are not purely energetic in origin.

The central role of AMPK in the lifespan extension suggests that energetic perturbation is still relevant to riboflavin depletion‐mediated longevity. Riboflavin depleted animals exhibit normal food consumption, normal TORC1 activation, and elevated triglyceride stores. Despite evidence for ample macronutrient availability, the animal experiences apparent energetic deficits with activation of AMPK during *rft‐1* RNAi. This begged the question as to whether the animal is activating dietary restriction (DR) pathways independent of true DR. The lifespan dependencies on FOXO/*daf‐16* and FOXA/*pha‐4,* as well as activation of canonical “starvation” reporters *acs‐2* and *bigr‐1*, provide evidence that the animal is utilizing these pathways in the context of micronutrient depletion only. Amino acid sensing and dietary restriction via essential nutrients such as methionine have been previously described to extend lifespan (Cabreiro et al., 2013). The depletion of canonical vitamin co‐factors, however, has proven deleterious in previous investigations. Depletion of biotin, B12 derivatives, and folate has previously shown to shorten lifespan (Austin et al., 2010; Bito et al., 2013). To the best of our knowledge, our work is the first to show that the depletion of a vitamin cofactor can mimic features of dietary restriction and extend lifespan using shared molecular mechanisms.

We anticipated that the relationships between the UPR^mt^, AMPK activity and *daf‐16* activation would be epistatic based on the hypothesized most proximal impact of having mitochondrial dysfunction. We noted, however, that FOXO nuclear localization occurs independent of AMPK but partially dependent on *atfs‐1*, and that upregulation of *sod‐3* is also dependent on *atfs‐1*. Transcriptional activation of *hsp‐6* is unexpectedly abrogated in *aak‐2* mutants during riboflavin depletion, suggesting that AMPK activity is necessary for potentiation of the UPR^mt^_._ This complex interplay of these dependencies hints that there is a concerted cellular response to reductions of the flavin cofactors. This creates the exciting opportunity to explore for flavin sensing molecular systems that converge on these key stress factors either upstream or downstream and may provide new avenues to activate pro‐longevity paradigms (Figure 6g).

The lipid phenotypes provide some clues to the nature of flavin sensing. Elevated triglyceride stores following riboflavin depletion is independent of canonical lifespan regulating pathways such as FOXO, AMPK, and TORC1. This decoupling of fat mass and lifespan suggests that the lipid phenotype may be regulated by enzymatic processing of lipids upstream of the energetic stress axes. The exceptions to this were the *atfs‐1* and *rict‐1* mutants. Phenotypes associated with UPR^mt^ activation are known to induce lipid accumulation (Kim, Grant et al. 2016, Yang, Li et al. 2022). Recent work has identified NHR‐80 as a key regulator of citrate sensing and lipid accumulation in the UPR^mt^ phenotype (Yang et al., 2022). The relevance of *atfs‐1* activity to *dve‐1* and *ubl‐5* function suggests that the UPR^mt^ may be instrumental in the communication of flavin depletion and related citric acid cycle disruptions on organismal energetics. The lack of fat mass increase in *rict‐1* mutants, which at baseline exhibit higher lipid content, suggests either a dependency or inability for riboflavin depletion to overcome the excess lipid accumulation associated with TORC2 knockout. TORC2 has been described as a nutritional sensor that regulates lipid biogenesis (Soukas et al., 2009), and it is entirely plausible that there are distinct inputs in mitochondrial energetics, mito‐stress, and TORC2 activation that are governed by flavin biology. Further investigation would be beneficial to identify whether TORC2 can directly sense changes in flavin levels, as this would have significant implications for the nutritional regulation of anabolic signals in senescence and cancer.

*rft‐1* RNAi does not impact developmental rate and metabolic phenotypes manifest most impressively at the young adult to adult day 1 transition. This is in parallel to the growth and development of the germline and the oocytes. This pattern suggests either that the larval stages are relatively resistant to riboflavin depletion, or, more likely, contain and accumulate sufficient flavin cofactors at time of egg lay and during early larval development (prior to *rft‐1* knockdown) to proceed through development normally. We surmise that somatic growth dilutes endogenous flavin cofactors, subsequently inducing the favorable effects of riboflavin deficiency uniquely in adulthood. Further, the development of the germline and riboflavin shunting into oocytes in late larval and early adult stages may prompt further, rapid riboflavin depletion, inducing the metabolic stress required to induce the phenotype identified in this work. This was particularly reflected in the germline RNAi deficient animals which revealed more pronounced lifespan extension compared with total body RNAi. It is worthy of mention that riboflavin deficiency leads to sterility, but this sterility is not accompanied by a decrease in germline stem cell numbers. Thus, effects on the germline are unlikely to be responsible for the shifts in lifespan and fat mass evident in riboflavin deficiency.

The presence of a post‐developmental fat increase with depletion of riboflavin suggests that acute depletion in adulthood has important impacts that are likely different from depletion starting at larval stages. The most likely etiology for these post‐developmental changes are alterations in enzymatic activity due to loss of flavin cofactors. The flavin cofactors are important for a wide variety of enzymes particularly those associated with oxido‐reductase functions including the fatty acid dehydrogenases. The “flavoproteome” is an established set of enzymes requiring FAD, FMN, or riboflavin to function (Lienhart et al., 2013). The impact of riboflavin depletion globally on the proteome is likely to produce stoichiometric shifts in key metabolites that will alter global physiology. Differential utilization of different dehydrogenases (branched chain vs. long chain) may also explain the unique lipid phenotype that is produced with riboflavin depletion. Using metabolomics, we identified other examples of likely enzymatic effects, with evidence of reductions in purine catabolism likely due to loss‐of‐function in xanthine dehydrogenase. Alterations in xanthine metabolism have been previously described as beneficial and lifespan extending (Gioran et al., 2019). The impact of riboflavin depletion on enzymatic processes is complex and there are likely to be both hormetic and harmful impacts of this process. Identifying the enzymatic pathways where riboflavin depletion provides beneficial versus detrimental impacts will provide new insights into mechanistic targets promoting longevity. We suggest that further investigations into the functions of the flavoproteome and flavin biology will serve to identify new therapeutic and investigational targets for the metabolism of aging and aging associated diseases.

## 4 EXPERIMENTAL METHODS

### 4.1 *C. elegans* genetics

Strains were maintained at 20°C on nematode growth medium (NGM) plates seeded with *E. coli* OP50. All experiments were conducted at 20°C unless otherwise specified. The following strains were utilized: wild type (N2 Bristol ancestral), NL3511 *ppw‐1*(*pk1425*), NL2098 *rrf‐1*(*pk1417*), *daf‐16*(*mgDF47*), TJ356 zls356[daf‐16p::daf‐16a/bGFP+rol‐6(su1006), CF1553 *muls84*[(pAD76)*sod‐3p*::GFP + *rol‐6*(*su1006*)], GR1318 *pdk‐1*(*mg142gf),* GR1310 *akt‐1*(*mg144gf*), RB754 *aak‐2*(*ok524*), VC3201 *atfs‐1*(*gk3094*), QC118 *atfs‐1*(*et18*), SJ4100 *zcls13*[*hsp‐6p*::GFP + *lin‐15*(+)], DMS303 *nls590*[fat‐7p::fat‐7::GFP + lin15(+)], VL749 *wwls24*[acdh‐1p::GFP + unc‐119(+)] MGH266 *rict‐1*(*mg451*), VC222 *raga‐1* (*ok386*), MGH559 *aak‐2*(*ok754*);*zls356*[*daf‐16p*::*daf‐16a/b*::GFP + *rol‐6*(*su1006*)], MGH249 *alxls19* [*bigr‐1*::*bigr‐1*::mRFP3‐HA;*myo‐2p*::GFP], WBM392 *Is*[*Pacs‐2::GFP + rol‐6(su1006)*]*,* MGH430 *rsks‐1(alx48* humanized S6K hydrophobic motif). MGH113 *nhr‐49*(*nr2041*), CB4037 *glp‐1*(*e2141*), DA465 *eat‐2*(*ad465*), WBM60 uthls248[*aak‐2p::aak‐2(genomic aa1‐321)::GFP::unc‐54 3′UTR + myo‐2p::tdTOMATO*], MGH600 *atfs‐1*(*gk3094*);*zls356*[*daf‐16p*::*daf‐16a/b*::GFP+ *rol‐6*(*su1006*)].

### 4.2 *E. coli* strains

Non‐RNAi experiments were all conducted on NGM plates containing *E. coli* OP50‐1 (CGC) as the food source and used 3–7 days after seeding. Cultures of *E. coli* OP50 were grown in Luria Broth (LB) for 15 h. at 37°C without shaking and seeded directly onto NGM plates. RNA interference experiments were conducted using *E. coli* HT115(DE3) bacteria (Ahringer library) as the food source. Clones were isolated from the primary RNAi library and plated on ampicillin/tetracycline plates. Individual clones were grown in LB broth for 15 h with shaking. Cultures were concentrated 1:5 and seeded directly onto NGM plates containing 5 mM isopropyl‐B‐D‐thiogalactopyranoside and 200 mg/ml carbenicillin. Plates were used 1–5 days after seeding. All RNAi clones were sequence verified prior to utilization. Riboflavin solution or vehicle was applied to the plate and allowed to dry for at least 30 min prior to seeding with animals.

### 4.3 Riboflavin treatment

Culture grade riboflavin (Sigma‐Aldrich) was dissolved in 50 mM NaOH to a concentration of 13.3 mM (5 mg/ml). Fully seeded plates were treated with 500ul riboflavin solution (final concentration 665 μM) and allowed to dry on the plate prior to worm placement. Vehicle plates were treated with 500 μl 50 mM NaOH solution.

### 4.4 Longevity assays

Lifespan analysis was conducted at 20°C except where indicated. Gravid adults were grown on NGM plates and isolated eggs were incubated overnight in M9 solution for hatching. Synchronized L1 animals were seeded unto RNAi plates and allowed to grow until the adult stage. Adult animals were subsequently transferred to fresh RNAi plates every other day until post‐reproductive stage where they were maintained on a single plate. Dead worms were counted daily or every other day. Statistical analysis for survival curves was performed using OASIS2 software (Han et al., 2016).

### 4.5 Development assays

Synchronized L1s were prepared via bleach prep and plated on RNAi plates containing empty vector or *rft‐1* RNAi grown at 20°C. Larvae were examined every 2 h starting 41 h after drop and scored for their transition to adulthood by the appearance of the vulvar slit.

### 4.6 Brood size

Fifty synchronized L1 animals of each strain were dropped on EV and *rft‐1* RNAi treated plates. Two days later, 2 young adult animals from each condition were dropped onto new EV and *rft‐1* RNAi plates, respectively. These animals were transferred every 2 days until the two adults from each condition became post‐reproductive. All animals on residual plates were counted once they reached L4/Young Adult to calculate brood size.

### 4.7 Pharyngeal pumping

Pumping rate was determined using a Sony camera attached to a Nikon SMZ1500 microscope that recorded 10 well fed day 1 adult animals per genotype. Pharyngeal contractions in 15 s time periods for 4 technical replicates were counted (by slowing video playback speed by 4×) for each animal using OpenShot and pumping rates per minute were calculated.

### 4.8 Food intake and activity assays

Food intake experiments were adapted from (Stuhr & Curran, 2020). Food intake was assessed in RNAi liquid media without antibiotics in flat‐bottom, optically clear 96‐well plates with 150 μl total volume. Age‐synchronized nematodes were seeded as L1 larvae and grown at 20°. 5‐Fluoro‐2′‐deoxyuridine (FUDR) was added 48 h after seeding at a final concentration of 0.12 mM. OD_600_ of each well was measured using a plate reader every 24 h starting at L1 stage and ending at day 5 of adulthood (168 h after dropping L1s). The fraction of animals alive was scored microscopically every day until the last day of the assay. Food intake per worms was calculated as bacterial clearance divided by worm number in well. Measurements were then normalized to the L4 to day 1 adult clearance rate for each condition. Lawn avoidance assays were conducted as described in (Stuhr & Curran, 2020). Bacteria were grown overnight in liquid culture of LB with corresponding antibiotics. The next day, bacteria were collected at the log phase, seeded onto RNAi plates at 5× concentration, dried, and allowed to grow overnight at 20°. L1s were dropped onto RNAi plates with each. Plates were checked 48 h later at the L4 stage, and the number of worms on and off food were counted. For size and movement assays, 30–50 worms were washed off of a plate in 50 μl of M9 with a M9 + triton coated P1000 tip and dropped onto an unseeded RNAi plate. The M9 was allowed to absorb and worms roamed on the unseeded plate for 1.5 h before imaging crawling. Crawling was imaged with the MBF Bioscience WormLab microscope and analysis was performed with WormLab version 2022. Worm crawling on the plate was imaged for 1 min for each condition at 7.5 ms. Worm crawling was analyzed with the software and only worms that moved for at least 90% of the time were included in the analysis.

### 4.9 Oil‐Red‐O and nile red staining

Lipid staining protocol was adapted from Escorcia et al. (2018). Adult day 1 animals were collected via washing and washed twice with M9 solution. Animals were then fixed with 40% isopropanol for 3 min with shaking. For oil‐red‐O staining, working solution of oil‐red‐O was generated from stock solution and fixed animals were stained in working solution for two hours. Animals were subsequently placed in M9 solution to remove excess stain and were imaged on a Leica Thunder multichannel microscope to generate composite images. For Nile red staining, Nile red working solution was generated by mixing 6 μl/Nile red stock solution in 1 ml isopropanol. Animals were stained in working solution for 2 h followed by 30 min of wash in M9 solution. Nile red imaging was performed on the Leica Thunder GFP setting with 10 ms exposure at 5× magnification.

### 4.10 Western blotting

Worms were isolated by washing with M9 buffer and centrifuged into a pellet. Worm lysates were prepared by adding RIPA buffer and proteinase inhibitor cocktail (Roche) followed by water bath sonication in a Diagenode Bioruptor XL 4 at maximum strength for 15 min. Lysates were cleared of debris via centrifugation at 21,000 *g* for 15 min at 4°C and supernatant was collected. Protein concentration as measured using the Pierce BCA Assay (Thermo Fisher). Lysate was subsequently mixed with 4X Laemmli buffer (Bio‐Rad) and boiled for 10 min. Samples were run on SDS‐PAGE protocol (Bio‐Rad) and transferred to nitrocellulose membrane via wet transfer at 100 V for 1 h. Immunoblotting was performed according to primary antibody manufacturer's protocols. Secondary antibody treatment utilized goat ‐anti‐rabbit HRP conjugate or goat‐anti‐mouse‐HRP conjugate (GE Healthcare) at 1:10,000 and 1:5000 dilutions, respectively. HRP chemiluminescence was detected via West‐Pico substrate (Thermo Pierce). The Western blot results shown are representative of at least two experiments. Primary antibodies used were the following:

Rabbit monoclonal anti‐Phospho‐AMPKα (Thr172), Cell Signaling Technology.
Rabbit monoclonal anti‐p70 Phospho‐S6 Kinase (Thr389), Cell Signaling Technology.
Mouse monoclonal anti‐Actin, Abcam.

### 4.11 Quantitative RT‐PCR

Worms samples were flash frozen in liquid nitrogen and kept in −80°C until RNA preparation. Samples were lysed through the use of metal beads and the Tissuelyser (Qiagen) Total RNA was extracted using RNAzol RT (Molecular Research Center) according to manufacturer instructions. RNA was treated with RNAse free DNAse prior to reverse transcription with the Quantitect reverse transcription kit (Qiagen). Quantitative RT‐PCR was conducted in triplicate using a Quantitect SYBR Green PCR reagent (Qiagen) following manufacturer instructions on a Bio‐Rad CFX96 Real‐Time PCR system (Bio‐Rad) Expression levels of tested genes were presented as normalized fold changes to the mRNA abundance of control genes indicated in the figures by the δδCt method.

The primers used for the qPCR are as follows:

*rft‐1* forward: GCTATTGTTCAGATCGCGTGC.
*rft‐1* reverse: CAGAGACCCAATTGACAAATACATGC.
*rft‐2* forward: CGGGAGTTGTTCAGATCGCT.
*rft‐2* reverse: GAGTCCCAGTTGACAACAGCA.
*rfk* forward: TGTTGGAAAAAGAAACGAAAGAA.
*rfk* reverse: TCGATTAAAATTCGGTAACAACG.
*flad‐1* forward: TGCCTGGAGTTCCAAAATTC.
*flad‐1* reverse: GAAGGGCTGGGTGTTTTACA.
*C07G1.7* forward: GCTGAAGAAGCTTCAACCGTAG.
*C07G1.7* reverse: TCTCGTGTCAATTCCGGTCT.
*hrg‐9* forward: TGGAATATTGAGTGGCGTTG.
*hrg‐9* reverse: CCTCCTCTACTTGGTGCATGT.
*cdr‐2* forward: CGAGCCTCATTTGGAAAGAA.
*cdr‐2* reverse: GCATCTGCCGCTGTAACTTT.
*sod‐3* forward: GCAATCTACTGCTCGCACTG.
*sod‐3* reverse: TGCATGATTTCATGGCTGAT.
*hsp‐6* forward: CGAAGACCCAGAGGTTCAAA.
*hsp‐6* reverse: AATGCTCCAACCTGAGATGG.

### 4.12 GC/MS lipid analysis

Lipid extraction and GC/MS of extracted, acid‐methanol derivatized lipids were performed as described previously (Pino & Soukas, 2020). Briefly, 10,000 synchronous mid‐L4 animals were sonicated with a probe sonicator on high intensity in a microfuge tube in 100–250 μl total volume. Following sonication, lipids were extracted in 3:1 methanol: methylene chloride following the addition of acetyl chloride in sealed borosilicate glass tubes, which were then incubated in a 75°C water bath for 1 h. Derivatized fatty acids and fatty alcohols were neutralized with 7% potassium carbonate, extracted with hexane, and washed with acetonitrile prior to evaporation under nitrogen. Lipids were resuspended in 200 μl of hexane and analyzed on an Agilent GC/MS equipped with a Supelcowax‐10 column as previously described (Pino et al., 2013). Fatty acids were indicated as the normalized peak area of the total of derivatized fatty acids detected in the sample, normalized by recovery of spiked‐in, standard phospholipid and triglyceride.

### 4.13 LC/MS metabolite analysis

Four biologic replicates of adult day 1 wild‐type worms treated either with empty vector or *rft‐1* RNAi were collected with M9 wash and frozen by liquid nitrogen into a worm pellet. Polar metabolites of homogenized worms were analyzed using a Thermo QExactive orbitrap mass spectrometer coupled to a Thermo Vanquish UPLC system, as previously described (Garratt et al., 2018). Bioactive lipids metabolites were profiled on the same system, as previous described (Lagerborg et al., 2019). Collected data were imported into the mzMine 2 software suite for analysis (version 2.53). Metabolites were annotated by using an in‐house library of commercially available standards. Please see supplemental methods for detailed methods. All mass integration values, normalized abundance values, significance testing scores, and pathway enrichment scores are included in this manuscript as Table S2.

### 4.14 Quantification and statistical analysis

All Western blotting quantifications were conducted on Bio‐Rad Image Lab. Intensity analysis for fluorescent images was performed on ImageJ. Statistical analyses were performed using Prism (GraphPad Software). The statistical differences between control and experimental groups were determined by two‐tailed students *t*‐test (two groups), one‐way ANOVA (more than two groups), two‐way ANOVA (two independent experimental variables), or log‐rank (survival analyses) as indicated in each figure legend, with numbers of samples indicated and corrected *p* values < 0.05 considered significant.

### 4.15 Fluorescence microscopy

DIC, brightfield and fluorescence Imaging of animals was performed utilizing the Leica Thunder microscopy system. Animals were picked onto a slide containing agar and 2.5 mM levimasole solution. Imaging was performed within 5 min of slide placement. GFP and RFP Images were taken at 10 ms exposure at 30% FIM and at 5X magnification, unless otherwise specified. Fluorescence intensity for quantification was calculated utilizing ImageJ software. For signal intensity experiments, quantification was performed on 20 worms (10 worms of two biological replicates).

### 4.16 Two‐photon and fluorescence lifetime imaging

Wild type and mutant *C. elegans* were immobilized for fluorescence imaging using a previously proposed protocol (Kim et al., 2013). Endogenous two‐photon excited fluorescence (TPEF) images of *C. elegans* were acquired using a laser scanning microscope (Leica TCS SP8, Wetzlar, Germany) equipped with a femtosecond laser (Insight Deep See, Spectra Physics, Mountain View, CA). Fluorescence lifetime images (512 × 512 pixels) of *C. elegans* were acquired using the same excitation and emission settings as for intensity NAD(P)H and FAD images and a PicoHarp 300 time‐correlated single photon counting unit (PicoQuant, Berlin, Germany) integrated in the Leica SP8 system. Please see Supplemental methods for further details on methods and analysis.

### 4.17 Stimulated Raman scattering imaging

Stimulated Raman scattering (SRS) images of *C. elegans* were acquired using a laser scanning confocal microscope (Leica TCS SP8, Wetzlar, Germany) equipped with a picosecond NIR laser (picoEmerald, APE, Berlin, Germany). SRS images were acquired in the wavenumber range of 2798 to 3103 cm^−1^ with an interval of 6 cm^−1^. The Nd:VAN 1064.2 nm output was used as the SRS Stokes beam and the OPO beam tuned from 800 nm to 820 nm with step size of 0.4 nm was used as the pump laser. SRS images (620 × 620 microns × 51 wavenumbers, 512 × 512 pixels × 51 wavenumbers, 0.75 zoom) were acquired using a water immersion objective HCX IRAPO L 25×/0.95 NA with pixel dwell time of 4.9 μs. The pixels corresponding to regions occupied by *C. elegans* were identified by implementing a global threshold of 300 (intensities ranged from 0 to 800). The SRS spectrum of each remaining pixel was normalized by the maximum SRS value of the entire field spectral image. To estimate the relative unsaturation level of the lipids in *C. elegans*, a ratio metric approach was adapted (Freudiger et al., 2011; Nieva et al., 2012; Potcoava et al., 2014; Verma & Wallach, 1977). Specifically, the ratio of the area under the SRS spectrum for wavenumbers spanning 2991 and 3022 cm^−1^ and wavenumbers spanning 2830 and 2870 cm‐1was estimated to represent the relative unsaturation levels (Freudiger et al., 2011, Nieva et al., 2012, Potcoava et al., 2014, Verma & Wallach, 1977). Both fluorescence and SRS images were calibrated for laser power before analysis.

## AUTHOR CONTRIBUTIONS

AY, LM, DB, and AAS conceptualized the study. AY, LM, DB, and SE performed methodology. AY, LM, DB, and SE were involved in validation. AY, LM, SE, DB, SL, YZ, AA, LC, NS, and AAS contributed to investigation. AY, EG, KD, MJ, IG, and AAS performed formal analyses. AY and AAS were involved in writing. AY, FA, SE, LM, YZ, SL, LC, AA, DB, NS, EG, MJ, IG, and AAS were involved in review and editing. AY, IG, SPC, and AAS contributed to funding.

## Conflict of Interest

The authors report no competing interests.

## Supporting information


**Figure S1** **–S5**
Appendix S1

Dataset S1

Dataset S2

## Data Availability

The data that support the findings of this study are available from the corresponding author upon reasonable request.

## References

[acel13718-bib-0001] Alonzo, C. A. , Karaliota, S. , Pouli, D. , Liu, Z. , Karalis, K. P. , & Georgakoudi, I. (2016). Two‐photon excited fluorescence of intrinsic fluorophores enables label‐free assessment of adipose tissue function. Scientific Reports, 6, 31012. 27491409 10.1038/srep31012 PMC4974509

[acel13718-bib-0002] Austin, M. U. , Liau, W. S. , Balamurugan, K. , Ashokkumar, B. , Said, H. M. , & LaMunyon, C. W. (2010). Knockout of the folate transporter folt‐1 causes germline and somatic defects in *C. elegans* . BMC Developmental Biology, 10, 46. 20441590 10.1186/1471-213X-10-46 PMC2874772

[acel13718-bib-0003] Bennett, C. F. , Vander Wende, H. , Simko, M. , Klum, S. , Barfield, S. , Choi, H. , Pineda, V. V. , & Kaeberlein, M. (2014). Activation of the mitochondrial unfolded protein response does not predict longevity in Caenorhabditis elegans. Nature Communications, 5, 3483. 10.1038/ncomms4483 PMC3984390 24662282

[acel13718-bib-0004] Biswas, A. , Elmatari, D. , Rothman, J. , LaMunyon, C. W. , & Said, H. M. (2013). Identification and functional characterization of the *Caenorhabditis elegans* riboflavin transporters rft‐1 and rft‐2. PLoS One, 8(3), e58190. 23483992 10.1371/journal.pone.0058190 PMC3590142

[acel13718-bib-0005] Bito, T. , Matsunaga, Y. , Yabuta, Y. , Kawano, T. , & Watanabe, F. (2013). Vitamin B12 deficiency in *Caenorhabditis elegans* results in loss of fertility, extended life cycle, and reduced lifespan. FEBS Open Bio, 3, 112–117. 10.1016/j.fob.2013.01.008 PMC3668511 23772381

[acel13718-bib-0006] Cabreiro, F. , Au, C. , Leung, K. Y. , Vergara‐Irigaray, N. , Cocheme, H. M. , Noori, T. , Weinkove, D. , Schuster, E. , Greene, N. D. , & Gems, D. (2013). Metformin retards aging in *C. elegans* by altering microbial folate and methionine metabolism. Cell, 153(1), 228–239. 23540700 10.1016/j.cell.2013.02.035 PMC3898468

[acel13718-bib-0007] Dillin, A. , Hsu, A. L. , Arantes‐Oliveira, N. , Lehrer‐Graiwer, J. , Hsin, H. , Fraser, A. G. , Kamath, R. S. , Ahringer, J. , & Kenyon, C. (2002). Rates of behavior and aging specified by mitochondrial function during development. Science, 298(5602), 2398–2401. 12471266 10.1126/science.1077780

[acel13718-bib-0008] Dipti, S. , Childs, A. M. , Livingston, J. H. , Aggarwal, A. K. , Miller, M. , Williams, C. , & Crow, Y. J. (2005). Brown‐Vialetto‐Van Laere syndrome; variability in age at onset and disease progression highlighting the phenotypic overlap with Fazio‐Londe disease. Brain Dev, 27(6), 443–446. 16122634 10.1016/j.braindev.2004.10.003

[acel13718-bib-0009] Durieux, J. , Wolff, S. , & Dillin, A. (2011). The cell‐non‐autonomous nature of electron transport chain‐mediated longevity. Cell, 144(1), 79–91. 21215371 10.1016/j.cell.2010.12.016 PMC3062502

[acel13718-bib-0010] Escorcia, W. , Ruter, D. L. , Nhan, J. , & Curran, S. P. (2018). Quantification of Lipid Abundance and Evaluation of Lipid Distribution in Caenorhabditis elegans by Nile Red and Oil Red O Staining. Journal of Visualized Experiments, 133, 57352. 10.3791/57352 PMC5931440 29553519

[acel13718-bib-0011] Feng, J. , Bussiere, F. , & Hekimi, S. (2001). Mitochondrial electron transport is a key determinant of life span in *Caenorhabditis elegans* . Developmental Cell, 1(5), 633–644. 11709184 10.1016/s1534-5807(01)00071-5

[acel13718-bib-0012] Freudiger, C. W. , Min, W. , Holtom, G. R. , Xu, B. , Dantus, M. , & Xie, X. S. (2011). Highly specific label‐free molecular imaging with spectrally tailored excitation stimulated Raman scattering (STE‐SRS) microscopy. Nature Photonics, 5(2), 103–109. 23015809 10.1038/nphoton.2010.294 PMC3454352

[acel13718-bib-0013] Gandhimathi, K. , Karthi, S. , Manimaran, P. , Varalakshmi, P. , & Ashokkumar, B. (2015). Riboflavin transporter‐2 (rft‐2) of Caenorhabditis elegans: Adaptive and developmental regulation. Journal of Biosciences, 40(2), 257–268. 25963255 10.1007/s12038-015-9512-x

[acel13718-bib-0014] Garratt, M. , Lagerborg, K. A. , Tsai, Y. M. , Galecki, A. , Jain, M. , & Miller, R. A. (2018). Male lifespan extension with 17‐alpha estradiol is linked to a sex‐specific metabolomic response modulated by gonadal hormones in mice. Aging Cell, 17(4), e12786. 29806096 10.1111/acel.12786 PMC6052402

[acel13718-bib-0015] Gioran, A. , Piazzesi, A. , Bertan, F. , Schroer, J. , Wischhof, L. , Nicotera, P. , & Bano, D. (2019). Multi‐omics identify xanthine as a pro‐survival metabolite for nematodes with mitochondrial dysfunction. The EMBO Journal, 38(6), e99558. 30796049 10.15252/embj.201899558 PMC6418696

[acel13718-bib-0016] Greer, E. L. , Dowlatshahi, D. , Banko, M. R. , Villen, J. , Hoang, K. , Blanchard, D. , Gygi, S. P. , & Brunet, A. (2007). An AMPK‐FOXO pathway mediates longevity induced by a novel method of dietary restriction in *C. elegans* . Current Biology, 17(19), 1646–1656. 17900900 10.1016/j.cub.2007.08.047 PMC2185793

[acel13718-bib-0017] Han, S. , Schroeder, E. A. , Silva‐Garcia, C. G. , Hebestreit, K. , Mair, W. B. , & Brunet, A. (2017). Mono‐unsaturated fatty acids link H3K4me3 modifiers to *C. elegans* lifespan. Nature, 544(7649), 185–190. 28379943 10.1038/nature21686 PMC5391274

[acel13718-bib-0018] Han, S. K. , Lee, D. , Lee, H. , Kim, D. , Son, H. G. , Yang, J. S. , Lee, S. V. , & Kim, S. (2016). OASIS 2: Online application for survival analysis 2 with features for the analysis of maximal lifespan and healthspan in aging research. Oncotarget, 7(35), 56147–56152. 27528229 10.18632/oncotarget.11269 PMC5302902

[acel13718-bib-0019] Kim, E. , Sun, L. , Gabel, C. V. , & Fang‐Yen, C. (2013). Long‐term imaging of Caenorhabditis elegans using nanoparticle‐mediated immobilization. PLoS One, 8(1), e53419. 23301069 10.1371/journal.pone.0053419 PMC3536676

[acel13718-bib-0020] Kim, H. E. , Grant, A. R. , Simic, M. S. , Kohnz, R. A. , Nomura, D. K. , Durieux, J. , Riera, C. E. , Sanchez, M. , Kapernick, E. , Wolff, S. , & Dillin, A. (2016). Lipid biosynthesis coordinates a mitochondrial‐to‐cytosolic stress response. Cell, 166(6), 1539–1552 e1516. 27610574 10.1016/j.cell.2016.08.027 PMC5922983

[acel13718-bib-0021] Kimura, K. , Tanaka, N. , Nakamura, N. , Takano, S. , & Ohkuma, S. (2007). Knockdown of mitochondrial heat shock protein 70 promotes progeria‐like phenotypes in caenorhabditis elegans. The Journal of Biological Chemistry, 282(8), 5910–5918. 17189267 10.1074/jbc.M609025200

[acel13718-bib-0022] Lagerborg, K. A. , Watrous, J. D. , Cheng, S. , & Jain, M. (2019). High‐throughput measure of bioactive lipids using non‐targeted mass spectrometry. Methods in Molecular Biology, 1862, 17–35. 30315457 10.1007/978-1-4939-8769-6_2

[acel13718-bib-0023] Lee, S. S. , Kennedy, S. , Tolonen, A. C. , & Ruvkun, G. (2003). DAF‐16 target genes that control C. elegans life‐span and metabolism. Science, 300(5619), 644–647. 12690206 10.1126/science.1083614

[acel13718-bib-0024] Lienhart, W. D. , Gudipati, V. , & Macheroux, P. (2013). The human flavoproteome. Archives of Biochemistry and Biophysics, 535(2), 150–162. 23500531 10.1016/j.abb.2013.02.015 PMC3684772

[acel13718-bib-0025] Liu, Z. , Pouli, D. , Alonzo, C. A. , Varone, A. , Karaliota, S. , Quinn, K. P. , Munger, K. , Karalis, K. P. , & Georgakoudi, I. (2018). Mapping metabolic changes by noninvasive, multiparametric, high‐resolution imaging using endogenous contrast. Science Advances, 4(3), eaap9302. 29536043 10.1126/sciadv.aap9302 PMC5846284

[acel13718-bib-0026] Mansoorabadi, S. O. , Thibodeaux, C. J. , & Liu, H. W. (2007). The Diverse Roles of Flavin CoenzymesNature's Most Versatile Thespians. The Journal of Organic Chemistry, 72(17), 6329–6342. 17580897 10.1021/jo0703092 PMC2519020

[acel13718-bib-0027] Massey, V. (1995). Introduction: flavoprotein structure and mechanism. The FASEB Journal, 9(7), 473–475. 7737454 10.1096/fasebj.9.7.7737454

[acel13718-bib-0028] Moriyama, Y. (2011). Riboflavin transporter is finally identified. Journal of Biochemistry, 150(4), 341–343. 21810912 10.1093/jb/mvr095

[acel13718-bib-0029] Nabokina, S. M. , Subramanian, V. S. , & Said, H. M. (2012). Effect of clinical mutations on functionality of the human riboflavin transporter‐2 (hRFT‐2). Molecular Genetics and Metabolism, 105(4), 652–657. 22273710 10.1016/j.ymgme.2011.12.021 PMC3309148

[acel13718-bib-0030] Nieva, C. , Marro, M. , Santana‐Codina, N. , Rao, S. , Petrov, D. , & Sierra, A. (2012). The lipid phenotype of breast cancer cells characterized by Raman microspectroscopy: towards a stratification of malignancy. PLoS One, 7(10), e46456. 23082122 10.1371/journal.pone.0046456 PMC3474759

[acel13718-bib-0031] Panowski, S. H. , Wolff, S. , Aguilaniu, H. , Durieux, J. , & Dillin, A. (2007). PHA‐4/Foxa mediates diet‐restriction‐induced longevity of *C. elegans* . Nature, 447(7144), 550–555. 17476212 10.1038/nature05837

[acel13718-bib-0032] Paradis, S. , & Ruvkun, G. (1998). *Caenorhabditis elegans* Akt/PKB transduces insulin receptor‐like signals from AGE‐1 PI3 kinase to the DAF‐16 transcription factor. Genes & Development, 12(16), 2488–2498. 9716402 10.1101/gad.12.16.2488 PMC317081

[acel13718-bib-0033] Pino, E. C. , & Soukas, A. A. (2020). Quantitative profiling of lipid species in caenorhabditis elegans with gas chromatography‐mass spectrometry. Methods in Molecular Biology, 2144, 111–123. 32410029 10.1007/978-1-0716-0592-9_10

[acel13718-bib-0034] Pino, E. C. , Webster, C. M. , Carr, C. E. , & Soukas, A. A. (2013). Biochemical and high throughput microscopic assessment of fat mass in Caenorhabditis elegans. Journal of Visualized Experiments, 73, 50180. 10.3791/50180 PMC3944676 23568026

[acel13718-bib-0035] Potcoava, M. C. , Futia, G. L. , Aughenbaugh, J. , Schlaepfer, I. R. , & Gibson, E. A. (2014). Raman and coherent anti‐Stokes Raman scattering microscopy studies of changes in lipid content and composition in hormone‐treated breast and prostate cancer cells. Journal of Biomedical Optics, 19(11), 111605. 24933682 10.1117/1.JBO.19.11.111605 PMC4059341

[acel13718-bib-0036] Powers, H. J. (2003). Riboflavin (vitamin B‐2) and health. The American Journal of Clinical Nutrition, 77(6), 1352–1360. 12791609 10.1093/ajcn/77.6.1352

[acel13718-bib-0037] Qi, B. , Kniazeva, M. , & Han, M. (2017). A vitamin‐B2‐sensing mechanism that regulates gut protease activity to impact animal's food behavior and growth. eLife, 6, e26243. 28569665 10.7554/eLife.26243 PMC5478268

[acel13718-bib-0038] Schiavi, A. , Torgovnick, A. , Kell, A. , Megalou, E. , Castelein, N. , Guccini, I. , Marzocchella, L. , Gelino, S. , Hansen, M. , Malisan, F. , Condo, I. , Bei, R. , Rea, S. L. , Braeckman, B. P. , Tavernarakis, N. , Testi, R. , & Ventura, N. (2013). Autophagy induction extends lifespan and reduces lipid content in response to frataxin silencing in *C. elegans* . Experimental Gerontology, 48(2), 191–201. 23247094 10.1016/j.exger.2012.12.002 PMC3572394

[acel13718-bib-0039] Soo, S. K. , & Van Raamsdonk, J. M. (2021). High confidence ATFS‐1 target genes for quantifying activation of the mitochondrial unfolded protein response. microPublication Biology, 10, 17912. 10.17912/micropub.biology.000484 PMC8527333 34693215

[acel13718-bib-0040] Soukas, A. A. , Kane, E. A. , Carr, C. E. , Melo, J. A. , & Ruvkun, G. (2009). Rictor/TORC2 regulates fat metabolism, feeding, growth, and life span in *Caenorhabditis elegans* . Genes & Development, 23(4), 496–511. 19240135 10.1101/gad.1775409 PMC2648650

[acel13718-bib-0041] Spagnoli, C. , & De Sousa, C. (2012). Brown‐Vialetto‐Van Laere syndrome and Fazio‐Londe disease ‐ treatable motor neuron diseases of childhood. Developmental Medicine and Child Neurology, 54(4), 292–293. 22211384 10.1111/j.1469-8749.2011.04179.x

[acel13718-bib-0042] Stuhr, N. L. , & Curran, S. P. (2020). Bacterial diets differentially alter lifespan and healthspan trajectories in *C. elegans* . Communications Biology, 3(1), 653. 33159120 10.1038/s42003-020-01379-1 PMC7648844

[acel13718-bib-0043] Subramanian, V. S. , Subramanya, S. B. , Rapp, L. , Marchant, J. S. , Ma, T. Y. , & Said, H. M. (2011). Differential expression of human riboflavin transporters −1, −2, and −3 in polarized epithelia: a key role for hRFT‐2 in intestinal riboflavin uptake. Biochimica et Biophysica Acta, 1808(12), 3016–3021. 21854757 10.1016/j.bbamem.2011.08.004 PMC3196270

[acel13718-bib-0044] Van Gilst, M. R. , Hadjivassiliou, H. , & Yamamoto, K. R. (2005). A Caenorhabditis elegans nutrient response system partially dependent on nuclear receptor NHR‐49. Proceedings of the National Academy of Sciences of the United States of America, 102(38), 13496–13501. 16157872 10.1073/pnas.0506234102 PMC1201344

[acel13718-bib-0045] Verma, S. P. , & Wallach, D. F. (1977). Raman spectra of some saturated, unsaturated and deuterated C18 fatty acids in the HCH‐deformation and CH‐stretching regions. Biochimica et Biophysica Acta, 486(2), 217–227. 836854 10.1016/0005-2760(77)90018-2

[acel13718-bib-0046] Watson, E. , MacNeil, L. T. , Arda, H. E. , Zhu, L. J. , & Walhout, A. J. M. (2013). Integration of metabolic and gene regulatory networks modulates the *C. elegans* dietary response. Cell, 153(1), 253–266. 23540702 10.1016/j.cell.2013.02.050 PMC3817025

[acel13718-bib-0047] Wu, L. , Zhou, B. , Oshiro‐Rapley, N. , Li, M. , Paulo, J. A. , Webster, C. M. , Mou, F. , Kacergis, M. C. , Talkowski, M. E. , Carr, C. E. , Gygi, S. P. , Zheng, B. , & Soukas, A. A. (2016). An ancient, unified mechanism for metformin growth inhibition in *C. elegans* and cancer. Cell, 167(7), 1705–1718 e1713. 27984722 10.1016/j.cell.2016.11.055 PMC5390486

[acel13718-bib-0048] Wu, Z. , Senchuk, M. M. , Dues, D. J. , Johnson, B. K. , Cooper, J. F. , Lew, L. , Machiela, E. , Schaar, C. E. , DeJonge, H. , Blackwell, T. K. , & Van Raamsdonk, J. M. (2018). Mitochondrial unfolded protein response transcription factor ATFS‐1 promotes longevity in a long‐lived mitochondrial mutant through activation of stress response pathways. BMC Biology, 16(1), 147. 30563508 10.1186/s12915-018-0615-3 PMC6298126

[acel13718-bib-0049] Yamamoto, S. , Inoue, K. , Ohta, K. Y. , Fukatsu, R. , Maeda, J. Y. , Yoshida, Y. , & Yuasa, H. (2009). Identification and functional characterization of rat riboflavin transporter 2. Journal of Biochemistry, 145(4), 437–443. 19122205 10.1093/jb/mvn181

[acel13718-bib-0050] Yang, R. , Li, Y. , Wang, Y. , Zhang, J. , Fan, Q. , Tan, J. , Li, W. , Zou, X. , & Liang, B. (2022). NHR‐80 senses the mitochondrial UPR to rewire citrate metabolism for lipid accumulation in *Caenorhabditis elegans* . Cell Reports, 38(2), 110206. 35021096 10.1016/j.celrep.2021.110206

[acel13718-bib-0051] Yen, C. A. , Ruter, D. L. , Turner, C. D. , Pang, S. , & Curran, S. P. (2020). Loss of flavin adenine dinucleotide (FAD) impairs sperm function and male reproductive advantage in *C. elegans* . eLife, 9, e52899. 32022684 10.7554/eLife.52899 PMC7032928

[acel13718-bib-0052] Yonezawa, A. , Masuda, S. , Katsura, T. , & Inui, K. (2008). Identification and functional characterization of a novel human and rat riboflavin transporter, RFT1. American Journal of Physiology. Cell Physiology, 295(3), C632–C641. 18632736 10.1152/ajpcell.00019.2008

